# One treatment fits all: Effectiveness of a multicomponent cognitive behavioral therapy program in data-driven subtypes of perinatal depression

**DOI:** 10.1192/j.eurpsy.2021.398

**Published:** 2021-08-13

**Authors:** A. Waqas, A. Rahman

**Affiliations:** Institute Of Population Health, University of Liverpool, Liverpool, United Kingdom

**Keywords:** cluster analysis, Postpartum depression, phenotypic subtypes, heterogeneity

## Abstract

**Introduction:**

It has been well established that depressive disorders including perinatal depression are very heterogeneous, which partly explain the ineffectiveness of available treatments for many patients. Recent innovations in data science can help elucidate the nature of perinatal depression especially the heterogeneity in its presentation.

**Objectives:**

The present study aime to elucidate heterogeneous subtypes of PND and assess the effectiveness of a multicomponent cognitive behavioral therapy (CBT) across heterogenous subtypes of PND.

**Methods:**

This study was conducted in 2005 in two rural areas of Rawalpindi, Pakistan. Out of a total of 3,898 women, 903 pregnant women were identifed with PND (using DSM-IV) and randomly assigned to intervention and control group. Baseline assessments included interviewer admininstered Hamilton Depression Scale (HDS) and social risk factors. Follow-up assessments were conducted at 6 months and 12 months post-intervention. Principle component analysis was run to reduce dimensionality of the HDS. Two step cluster analysis was then run to elucidate subtypes of PND using the dimensional scores. Thereafter, effectiveness of CBT was compared across these subtypes of PND using multilevel modelling.

**Results:**

Principle component analysis revealed a four component solution for the Hamilton depression rating scale. Using these dimensional scores, cluster analysis (average silhouette= 0.5) revealed a parsimonius four cluster soultion of participants with mild PND symptoms (n=326); predominant sleep problems (n=311) c) predominant atypical symptoms (n=80) and d) comorbid depressive and anxiety symptoms (n=186). CBT yielded moderate effect sizes across all these subtypes of PND (cohen’s d > 0.8).

**Conclusions:**

Multicomponent CBT is effective across hetergeneous presentations of PND.

**Disclosure:**

No significant relationships.

